# Therapeutic potential of stem cells in addressing female infertility: recent progress and prospective developments

**DOI:** 10.3389/fmed.2026.1775277

**Published:** 2026-07-06

**Authors:** Yuyi Wu, Siying Chen, Ruotong Hao, Ruipeng Wang, Liandi Mai

**Affiliations:** 1 Department of Gynecology and Obstetrics, The Fifth People’s Hospital of Shunde (Longjiang Hospital of Shunde District), Foshan, China; 2 Guangzhou Medical University, Guangzhou, China; 3 Department of Gynecology and Obstetrics, The Second Affiliated Hospital of Dalian Medical University, Dalian, China; 4 Department of Reproductive Medicine, The Fifth People’s Hospital of Shunde (Longjiang Hospital of Shunde District), Foshan, China

**Keywords:** female infertility, mesenchymal stem cells, ovarian rejuvenation, regenerative medicine, stem cell therapy

## Abstract

Female infertility often results from ovarian insufficiency, endometrial damage, metabolic disorders, and treatment-induced reproductive decline. The regenerative benefits of emerging stem cell-based therapies are achieved through paracrine signaling, immunomodulation, angiogenesis, and anti-fibrotic effects. Preclinical and early clinical studies have shown that mesenchymal, pluripotent, embryonic, and reproductive progenitor cells, and their extracellular vesicles, can rescue ovarian function and improve endometrial receptivity, hormonal profiles, and follicular profiles. Although these are promising results, there are still issues with standardization, safety, and ethics. Further technological development and carefully designed clinical trials are the keys to successful translation. This review also demonstrates the underlying mechanisms, recent translational advances, and emerging technologies, as well as the need for standardized protocols and clinical validation.

## 1 Introduction

Infertility is experienced by 10–15 percent of women of reproductive age across the globe and is likely to rise due to late childbearing, environmental stressors, and lifestyle (1). Besides being an expensive experience in terms of emotional and socioeconomic concerns, infertility possesses one of the lowest success rates in the case of conventional therapy, as women with compromised ovarian reserve, uterine fibrosis, or permanent tissue damage do not have any chance of success with traditional treatment (2). Although assisted reproductive technologies overcome physiological barriers, they do not heal biological deficiencies.

The regenerative model of stem cell-based interventions is described by self-renewal, multi-lineage differentiation, and paracrine secretion. These treatments can control microenvironments, inhibit inflammation, trigger angiogenesis, and promote tissue repair in the ovaries and uterus, thereby bypassing the endodermal stage (3). Regenerative reproductive therapies are supported by the fact that stem cell biology has made great strides in the past two decades (4).

Mesenchymal stem cells (MSCs) are among the easily accessible stem cell types that have powerful immunomodulatory abilities (5). It has been further improved with induced pluripotent stem cells (iPSCs) and embryonic stem cells (ESCs), which have been utilized to develop artificial gametogenesis and tissue models (6). This literature review provides a mechanistic understanding, recent clinical developments, and perspectives on stem cell-based therapy in female infertility.

## 2 Types of stem cells applied in female infertility

### 2.1 Mesenchymal stem cells

The most commonly researched stem cell type for infertility is mesenchymal stem cells (MSCs) (5). These cells may be obtained from many sources, including but not limited to bone marrow, fat tissue, menstrual blood, umbilical cord, and amniotic membrane (6). The paracrine release of growth factors, extracellular vesicles and cytokines by ovarian and endometrial cells primarily mediates these therapeutic effects (7).

Mesenchymal stem cells suppress the apoptosis of granulosa cells, activate the primordial follicular activation, and improve the ovarian stromal activity. Umbilical cord-MSCs are angiogenic and have high angiogenic properties that can be used in the repair of endometrial tissue in Asherman syndrome (NCT03724617) (8). MSCs are specifically tailored to the uterine environment during menstruation, are highly proliferative, and can be used as autologous therapeutic tools (9). In recent years, some innovations in the field include genetic modification, preconditioning, and conjugation with biomaterials (10).

### 2.2 Pluripotent stem cells

Induced pluripotent stem cells are reprogrammed non-pluripotent cells that can differentiate into various lineages (11). Their use of autografts reduces their immunogenicity, and address ethical concerns. The iPSCs are also helpful for disease modeling, drug development, and the production of artificial gametes. They have been successfully differentiated into germ-cell-like and granulosa-like cells (12). In addition, a study has broken through the key formatting step of reproducing human germ cell development *in vitro* (13). It has demonstrated that germ line cells derived from pluripotent stem cells can complete epigenetic reprogramming, achieving the full process of *in vitro* reproduction from initiation to reprogramming completion. It has broken through this key bottleneck for the first time by identifying the BMP signaling pathway, providing a clear molecular framework for the subsequent use of pluripotent stem cells to study the complete process of human reproductive development.

### 2.3 Embryonic stem cells

Embryonic stem cells are undifferentiated, unlimited pluripotent cells that have been used to study the oogenesis and folliculogenesis pathways. These cells are able to differentiate into somatic and germ-like ovarian cell lines *in vitro* (14). Their potential to form teratomas and failure to achieve specific differentiation are problems in their clinical application that raise serious ethical concerns (15). However, research has been conducted to find safer alternatives to ESCs, including iPSCs.

### 2.4 Ovarian and endometrial progenitor cells

It has been suggested that ovarian germline germ cell stem cells can form oocyte-like cells; however, the future clinical use of these cells remains controversial (16). Periodically, the endometrial lining is regenerated by endometrial progenitor cells in the basalis layer, which are capable of repairing the damaged endometrium. This will qualify them as possible subjects in the treatment of Asherman syndrome and cases with chronic endometrial injury (17). Table 1 provides a comparative overview of the sources, mechanisms of action, targets, and new uses of stem cells in female infertility.

**TABLE 1:** Overview of stem cell types, mechanisms, targets, and clinical applications in female infertility.

Stem cell / technology	Source/derivation	Key mechanisms	Primary target condition(s)	Advantages	Limitations / risks	References
MSCs	Bone marrow	Paracrine support, anti-apoptotic effects	primary ovarian insufficiency (POI), diminished ovarian reserve (DOR)	Well-studied, safe	Limited differentiation	(18)
MSCs (adipose-derived)	Adipose tissue	Immunomodulation, repair signaling	Ovarian aging, polycystic ovary syndrome (PCOS)	Easy harvest	Donor variability	(19)
MSCs (umbilical cord)	Wharton’s jelly	Angiogenesis, stromal repair	Asherman, thin endometrium	Strong angiogenic effect	Allogeneic concerns	(20)
MSCs (menstrual blood)	Menstrual tissue	Endometrial regeneration	Thin endometrium	High proliferation, non-invasive	Early-stage data	(21)
MSCs (amniotic-derived)	Amniotic membrane	Anti-fibrotic action	Uterine scarring	Potent regenerative profile	Ethical considerations	(22)
IPSCs	Reprogrammed somatic cells	Differentiation into germ-cell-like cells	Genetic infertility	Autologous use possible	Tumorigenicity risk	(23)
ESCs	Blastocyst inner cell mass	Full pluripotency, germline potential	Oocyte regeneration	High differentiation potential	Ethical barriers	(24)
Ovarian progenitor cells	Ovary	Potential germline renewal	POI	Autologous potential	Controversial existence	(25)
Endometrial progenitor cells	Basalis layer	Endometrial reconstruction	Asherman syndrome	Natural regenerative role	Difficult isolation	(26)
MSC-derived exosomes	MSC secretome	MicroRNA delivery, anti-apoptotic	POI, thin endometrium	Cell-free, safe	Standardization needed	(27)
Exosome engineering	Modified MSC exosomes	Targeted signaling modulation	Ovarian and uterine dysfunction	Precision therapy	Technical complexity	(28)
Bioengineered ovarian scaffolds	Biomaterials + cells	3D structural support	Ovarian failure	Mimics natural ovary	Expensive, experimental	(29)
Bioengineered endometrial scaffolds	Collagen/hydrogel-based	Uterine tissue reconstruction	Severe endometrial damage	Supports implantation	Early-stage research	(30)
Gene-edited MSCs	CRISPR-enhanced MSCs	Enhanced paracrine output	POI, fibrosis	Higher potency	Gene-editing ethics	(31)
Artificial gametogenesis	iPSC/ESC → gamete-like cells	Oocyte formation *in vitro*	Loss of germ cells	Unlimited gamete supply	Safety unproven	(32)

### 2.5 A critical appraisal of stem cells

Mesenchymal stem cells primarily exert their effects through paracrine signaling, releasing growth factors and exosomes to optimize the microenvironment of the ovaries or uterus. Their action can be described as “repair” or “rejuvenation.” In contrast, induced iPSCs and ESCs possess the capacity to differentiate into germ-cell-like cells, with the goal of achieving complete replacement of damaged reproductive function.

Although iPSCs theoretically offer an unlimited source of gametes (via artificial gametogenesis), their tumorigenicity and genomic instability currently present insurmountable obstacles. Consequently, current clinical research favors the use of MSCs and their derivatives (such as exosomes), which boast higher safety profiles and fewer ethical controversies, despite their weaker direct differentiation potential.

### 2.6 Route of administration of stem cells

Delivery route substantially affects efficacy, homing, and safety, and this omission represents a significant gap in a review of this scope. According to existing research, whether in animal experiments or clinical trials, direct injection of stem cells into ovarian tissue (ovarian local injection) is considered the most effective way of administration (33). It is worth mentioning that a clinical study on the treatment of POI with human wool membrane cells (NCT02912104.) was administered through the ovarian artery and showed good results (34).

## 3 Mechanisms underlying stem cell-mediated reproductive repair

**FIGURE 1:**
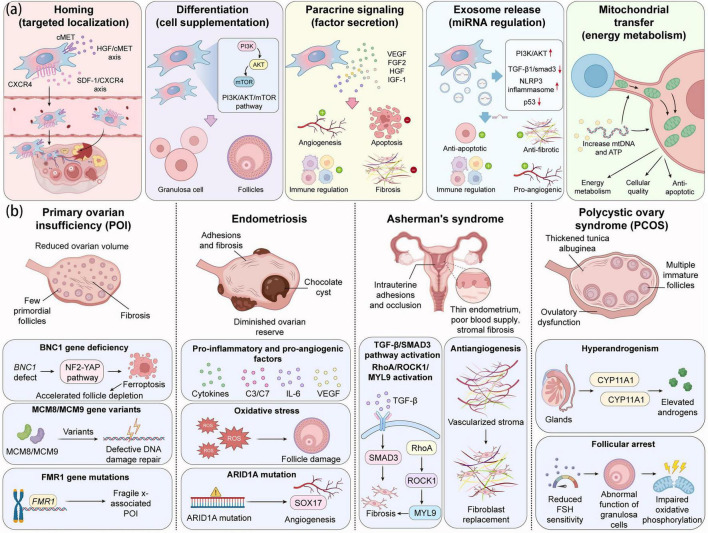
**(a)** Mechanisms and therapeutic areas of female infertility using stem cells. Key regenerative responses of stem cells (homing, differentiation, paracrine signaling, exosome release, and mitochondrial transfer) aid in ovarian repair (38). **(b)** Significant infertility disorders (POI, endometriosis, Asherman syndrome, PCOS) and their molecular changes compared to normal ovarian morphology (37).

The regeneration of reproductive activity depends mainly on the action of stem cells, via a cascade of interacting mechanisms that extends beyond direct differentiation. They mostly participate in paracrine signaling, immunomodulation, angiogenesis, and anti-fibrotic activities to augment the ovarian and uterine microenvironments (35) These mechanisms enhance follicle survival, hormone sensitivity, and endometrial tissue regeneration, which provides the biological basis for their clinical applicability in the treatment of infertility (36). Figure 1a shows the essential regenerative activities of stem cells, such as homing, differentiation, paracrine signaling, exosome release, and mitochondrial transfer, which regulate angiogenesis, apoptosis, fibrosis, and immune responses to restore ovarian function (37, 38).

### 3.1 Paracrine activation and anti-apoptotic effects

The initial mechanism through which stem cells stimulate repair during reproductive function is paracrine signaling (39). The mitotic activity of granulosa and stromal cells is stabilized by the release of growth factors, cytokines, and extracellular vesicles from stem cells, which alleviates apoptosis, enhances DNA repair, and normalizes mitochondrial activity (40). These processes support follicle survival, oocyte maturation, and enhanced ovarian responsiveness to gonadotropins (27). The uterine environment contains paracrine mediators that trigger epithelial and stromal growth, endogenous regenerative signaling, and help re-establish a functional endometrial structure (41).

### 3.2 Immunomodulation and anti-inflammatory regulation

One of the severe characteristics of different infertility diseases is a persistent inflammatory condition, including POI, PCOS, and endometrial damage. MSCs have good immunomodulatory capabilities (42). They suppress the release of pro-inflammatory cytokines and improve the release of anti-inflammatory cytokines (e.g., IL-10). They also prefer macrophage polarization to restore the M2 phenotype, thereby decreasing tissue destruction and enhancing repair (43). Immune system adaptations have been associated with the preservation of ovarian follicles, enhancement of endometrial receptivity, and restoration of physiological context, which facilitate conception.

### 3.3 Vascular remodeling

Adequate vascularization characterizes optimum ovarian and endometrial conditions. PDGF, VEGF, and other pro-angiogenic homodimers are secreted by the stem cells and mediate angiogenesis. This also enhances follicular growth by delivering oxygen and nutrients, leading to increased hormone exchange and improved stroma health in the ovaries as the vascular network expands. Thickness, glandular architecture, and receptivity can be enhanced by increased microvascular density in the endometrium, which is most evident in patients with a thin endometrium or uterine ischemia.

### 3.4 Anti-fibrotic mechanisms

The adverse effects of fibrosis on the physiological functions of the ovaries and uterus include loss of tissue elasticity, disturbances in vascular flow, and impaired cell communication (44). Stem cells play a critical role in reducing fibrosis by inhibiting TGF-β, which enhances profibrotic signaling, inhibits collagen synthesis, and promotes remodeling of the extracellular matrix (26). These anti-fibrotic effects are essential in situations such as Asherman syndrome and chronic inflammatory diseases, as it is highly important to reverse scarring of the reproductive tissue to restore normal tissue function (45, 46).

## 4 Stem cell applications in clinical conditions of female infertility

Ongoing research is being conducted on the use of stem cell-based therapies for various types of infertility, including ovarian dysfunction, absence of follicular support, and structural damage to the endometrium (47). These therapies rely on the regenerative, immunomodulatory, angiogenic, and anti-fibrotic effects of stem cells to address the underlying biological inadequacies, rather than relying on assisted reproductive technology (ART) for conception (48). Figure 1b illustrates the key reproductive diseases addressed by stem cell therapies: POI, endometriosis, Asherman syndrome, and PCOS, as well as the associated changes in the molecular state and their comparison with the usual ovarian physiology.

### 4.1 Ovarian dysfunction: POI, DOR, and age-related decline

Stem cell therapy has immense potential for treating diseases characterized by decreased ovarian reserve, such as POI and aging. MSCs can promote follicle survival, decrease apoptosis, preserve stromal integrity, and facilitate angiogenesis (7). A favorable hormone profile and increased follicle development have consistently been reported in preclinical studies. Preliminary clinical outcomes indicate that menstrual cyclic recovery, elevated anti-Müllerian hormone (AMH) levels, and enhanced ovarian response during *in vitro* fertilization (IVF) procedures should be beneficial in POI patients and even in older, poor responders (49, 50).

### 4.2 Endometrial damage: Asherman syndrome and thin endometrium

The significant features of Asherman syndrome include a thin endometrium, fibrosis, poor vascularization, and impaired endometrial regeneration. MSCs present in menstrual blood have been reported to accelerate healing, promote gland formation, and remodel blood vessels (9). Clinically, it has been noted to increase endometrial thickness, re-establish menstruation, and deliver successful pregnancies in non-responders to standard therapies (51). These findings show that stem cell treatment has significant potential for treating diseases related to the structure of the uterus through translation (10, 47).

### 4.3 Metabolic and hormonal disorders: polycystic ovary syndrome

Characteristics of PCOS are hormonal imbalance, anovulation, and long-term inflammation. MSCs also mediate inflammation, enhance insulin sensitivity, normalize androgen production, and restore physiological ovarian activity (8, 52). Animal studies have revealed that cyst development is decreased and ovulatory function is restored after MSC or MSC-related exosome administration, suggesting these agents can be used as an adjunctive treatment for severe or incurable PCOS. Mechanistically, MSCs can inhibit excessive autophagy and restore mitochondrial function in granulosa cells by regulating the PI3K/AKT/mTOR pathway (53), and can also prevent damage to oocytes by inhibiting the expression of WNT/α – catenin pathway (54). An RCT study (NCT05279768) suggests that umbilical cord-derived MSCs (UC-MSCs) may improve metabolic status by increasing adiponectin and improving insulin sensitivity in PCOS patients through immune regulation and epigenetic mechanisms (55).

### 4.4 Ovarian damage induced by chemotherapy and radiation

Radiotherapy and chemotherapy result in severe follicular depletion, stromal damage, and vascular damage in the ovarian (56, 57). MSCs have protective and regenerative functions that reduce oxidative stress, repair DNA, and regenerate blood vessels (58). It has been demonstrated that the mechanisms of hormone production and follicle count may also be restored through the aid of MSCs treatment in preclinical studies (59, 60). The above results contribute to the application of stem-cell treatment in maintaining fertility among cancer patients.

## 5 Stem cell-derived exosomes as a cell-free alternative

Stem cell exosomes have rapidly become an effective cell-free treatment. They may offer many of the therapeutic benefits of stem cell transplantation without the safety and regulatory challenges that stem cell transplantation entails. These small vesicles contain bioactive molecules linked to tissue repair, offering a viable and scalable solution for regenerative medicine (61).

### 5.1 Biological properties and mechanisms of action

The secretions in stem cell exosomes (protein-, lipid-, and RNA-containing) mediate apoptosis, oxidative stress, angiogenesis, and immune responses. Their lipid bilayer protects the cargo during circulation and during endometrial uptake by ovarian cells (62). The products of these interactions are cell survival, tissue remodeling, and better reproductive activity, with no cell engraftment or growth. They can also be used in clinical translation due to their good safety profile since they have not shown any signs of tumorigenicity or immunogenicity (63).

### 5.2 Therapeutic impact on ovarian and endometrial regeneration

**FIGURE 2:**
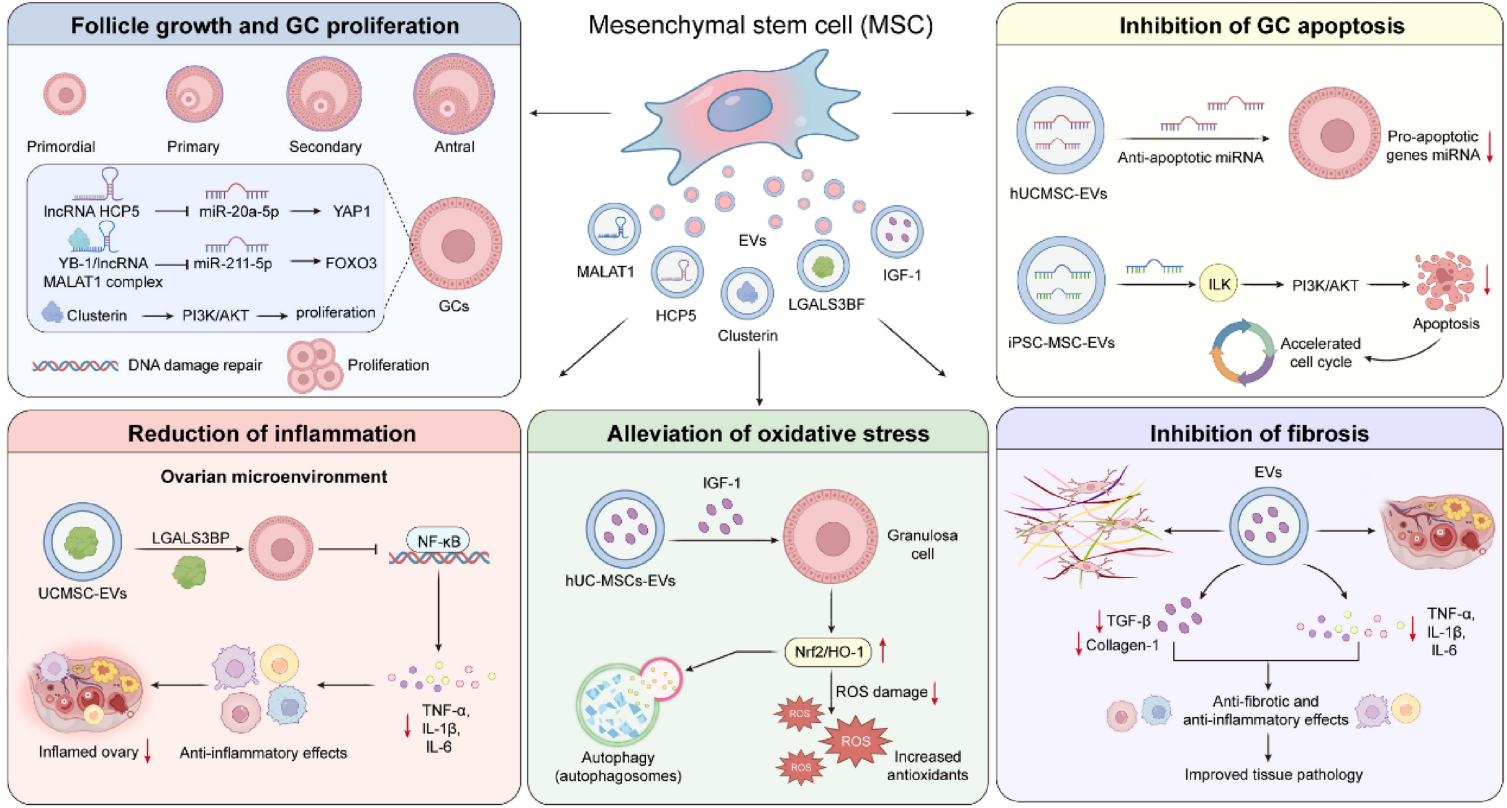
MSC-derived extracellular vesicles (EVs): mechanisms of ovarian repair. EVEs from MSCs release bioactive molecules and microRNAs to stimulate follicular growth and granulosa cell proliferation, and to decrease apoptosis, inflammation, oxidative stress, and fibrosis in POI (65).

Exosomes secreted by MSCs have shown great potential in restoring primary models of ovarian insufficiency. These effects include increased follicle number, restoration of hormone secretion, and granulosa cell death. Exosomes promote stromal and epithelial proliferation, angiogenesis, and activate repair pathways essential in the uterine environment, including the PI3K/AKT and ERK pathways. These interventions help increase endometrial thickness, organization, and receptivity, which may have therapeutic effects in conditions such as Asherman syndrome and in thin endometria that would otherwise be unresponsive to conventional treatment approaches (64). Figure 2 illustrates the mechanistic aspects of MSC-derived extracellular vesicles in the restoration of ovarian function, including their microRNA cargo, signaling pathways, and downstream effects in granulosa cells, inflammation, angiogenesis, oxidative stress, and fibrosis (65).

### 5.3 Clinical advantages and future development

Exosomes offer several advantages over cell-based therapies, including easy storage, high-standard production, low immunogenicity, and the ability to undergo repeat dosing. They have a strong regulatory profile, qualifying them as first-line candidates for reproductive regenerative medicine. The latest trends have focused on designing more resilient exosomes to prevent degradation, such as attaching microRNAs of interest or target ligands to surfaces, to enable precision, tissue-specific therapies (66). Exosomes will inevitably become an integral part of the treatment of ovarian and endometrial dysfunction in the future.

## 6 Clinical trials and emerging translation

Exosome-based therapies are emerging as the best option for clinical translation because of their ease of storage, ease of standard manufacturing, low immunogenicity, and potential for repeated administration (67). There is already a history of early research on engineered exosomes pulsed with targeted ligands or microRNAs to enhance therapeutic targeting. Exosomes are yet another promising development in the regenerative approach to ovarian and endometrial dysfunction, as technologies are being developed for official clinical trials (68).

### 6.1 Early clinical findings and therapeutic outcomes

Initial clinical trials (NCT03033277) involving intrauterine or intravaginal MSCs injection have shown that they rescued the ovarian function of POI patients.(69). Women with POI, reduced ovarian reserve, reduced anti-AMH levels, or improved IVF fertility rates have noted the recovery of menstruation, increased rates of anti-AMH, or improved IVF pregnancy (70). Embryo implantation has been successfully induced using stem cell-based therapy in cases of severe endometrial damage. Such findings are only applicable to small cohorts but are indicative of strong therapeutic potential (51).

### 6.2 Current limitations and need for standardization

Although positive results have been received in relation to preliminary data, the research studies conducted are crippled by methodological inadequacy. Most trials lacked randomization, a control group, and consistent patient selection criteria. Differences in stem cell sources, preparation, dose, and mode of delivery complicate comparisons of study outcomes and affect the reproducibility of results. In addition, the brief follow-up process did not provide sufficient information regarding the sustainability of safety and responsiveness. Evidence-based production, standardized production, GMP-compliant production, validated potency assays, and standardized clinical protocols are required to enhance evidence-based production.

### 6.3 Future directions for clinical translation

Major multicenter randomized trials with clearly defined primary outcomes, long-term follow-up, and strict adherence to reproductive, obstetric, and neonatal outcomes are required to establish stem cell therapy as a standard practice. Biomarkers will also be used to contribute to responsible translation and long-term safety monitoring through registries. A synergistic method that combines clinicians, researchers, and regulatory agencies can help develop stem cell-based therapies as a supplement or alternative to modern fertility treatments.

## 7 Ethical, Regulatory, and safety considerations

Infertility treatments involving stem cell-based therapies have massive ethical, safety, and regulatory issues. Due to the potential reproductive effects that such interventions can have on future generations of children, it is inevitable to establish sufficient management and clinical practice principles to conduct such interventions safely and responsibly (71).

### 7.1 Ethical issues and emerging technologies

The moral issues that arise include the procurement of stem cells, consent of the donors, and the just distribution of treatment. The use of ESCs is controversial because of their derivation from embryos; however, adult and perinatal stem cells must be regulated to prevent commercialization and abuse. The promotion of new technologies, such as artificial gametogenesis, gene editing, and engineered exosomes, introduces new complications related to genetic parenthood, germline modification, and societal implications. Such difficulties provide the answer to the question because they create flexible norms of morality that can adapt to the flow of scientific discovery (72).

### 7.2 Safety risks and long-term monitoring

Patient safety is the most significant concern in stem cell reproductive therapy. Although MSCs are generally safe, they carry risks, including contamination during processing, immune responses, and unwanted differentiation. However, iPSCs and ESCs also have other risks, including tumorigenesis and genomic instability (73). The ability to adjust for ovarian aging, hormonal phenotype, pregnancy outcomes, and offspring well-being is necessary to determine delayed or multigenerational outcomes, along with long-term follow-up and longitudinal registries.

### 7.3 Regulatory oversight and standardization

In stem cell therapies, many regulatory policies must be adhered to, such as gold-standard manufacturing practices, potency assessment validation, and scientifically designed clinical studies. The high variation in cell sourcing, culture, and cell-delivery methods poses a challenge to inter-study reproducibility. Similarly, exosome-based therapies should be standardized in terms of purification and characterization to ensure consistent treatment quality. International regulatory standards will be standardized, thereby making clinical translation safer and more efficient.

### 7.4 Balancing innovation with responsible practice

It is the dilemma that reproductive medicine must grapple with: the thin line between patient safety and science. The untested treatment will not only be detrimental to the patients, but it will also cause the development of a negative attitude toward the drug in the patients. Meanwhile excess regulation has the propensity of forcing the underlying payoffs to the bottom. The stem cell treatments ought to be put on the check, and accountability ought to be achieved personally, which is grounded on the elements of security, fairness and scientific integrity.

## 8 Future prospects and innovative directions

The recent developments of the impact on stem cell-based infertility interventions have been led by regenerative biology, biomaterials, gene editing, and personalized medicine. The innovations will lead to the increased efficacy of the therapeutic interventions, their safety, and the variety of choices to women with so-called incurable conditions.

### 8.1 Scientific advances driving regenerative reproductive medicine

This depends on a detailed appreciation of tissue niches, follicular stimulation, and the recovery processes that restore ovarian and endometrial tissues in the future. Single-cell sequencing, lineage tracking, and advanced imaging techniques are regenerative identification methods that have been defined and enable the development of targeted procedures. Moreover, artificial gametogenesis is another significant conceptual development, as oocytes can be generated from pluripotent cells. However, this is a severe problem that raises important biological and ethical questions and cannot be applied in a healthcare context.

### 8.2 Genetic and molecular engineering for enhanced therapies

The current focus on gene-editing technology, namely, CRISPR-Cas systems, can help heal heritable infertility disorders and optimize therapeutic stem cells, conditioning them to survive, become paracrine, and become stress-resistant (74). Exosomes, which are modified to carry specific ligands or microRNAs, can provide more defined regenerative cues. These approaches can be practical, but they require close attention because the side effects of genetic modification, including effects on non-targets and overall impact, should be considered (75).

### 8.3 Biomaterials, 3D bioprinting, and tissue engineering

Recent advances in biomaterials (hydrogels, scaffolds, three-dimensional printed structures) and the use of flexible electromechanical materials with the potential to detect deformation and mechanical responses, also enhance the viability of those stem cells and the capability to use three-dimensional printing to direct tissues (76, 77). A possible solution that was suggested to control the desperate annihilation of reproductive tissues was the bioengineering of the ovarian and endometrial systems. Such technologies can also be superior to traditional cell therapy, which provides structurally and functionally optimized tissue replacements for degenerative illnesses (78).

### 8.4 Personalization, integration with ART, and remaining challenges

The potential applications of personalized regenerative medicine enabled by genomic profiling and artificial intelligence include identifying the best candidates, predicting treatment response, and determining the optimal treatment regimens. Regenerative therapies will be used in conjunction with existing assisted reproductive technologies to enhance ovarian responsiveness and endometrial receptivity in complicated cases. The advancement in wearable sensing and feedback potentials accelerates this trend since it allows to constantly monitor and optimize the reproductive health interventions in people on an individual basis (79, 80). With the development, there are still some underlying issues including tissue stabilization, repeated fibrosis, standardization of the manufacturing process and ethical issues surrounding gene editing and the generation of unnatural gametes.

However, the ethical governance framework of International Society for Stem Cell Research (ISSCR) is dynamic and continuously evolving. Recently, specialized supplementary guidance documents have been released for research on stem cell-based embryo models (SCBEMs), reflecting their up-to-date characteristics. Overall, the ISSCR 2021 guidelines provide a timely, comprehensive, and forward-looking ethical framework for the rapidly developing field of biomedical research. By categorizing and managing research, it cleverly seeks a balance between scientific exploration and ethical responsibility, aiming to ensure that scientific progress benefits humanity in a responsible and ethical manner.

## 9 Conclusion

Therapeutic interventions for female infertility based on stem cells are an innovative breakthrough in the field of reproductive medicine, which can provide regenerative therapy where traditional treatment drugs fail to work. Various types of stem cells and their exosomes have shown the ability to restore ovarian functionality, heal endometrial tissue, as well as treat metabolic or treatment-induced reproductive injury via paracrine signaling, immunomodulation, angiogenesis, and anti-fibrotic effects. The initial clinical results are encouraging; however, the wider clinical usage still requires addressing the major issues of standardization, safety, long-term follow-up, and ethical control. With medical therapies still developing with the advancements in biomaterials, genetic engineering, and personalized medicine, stem cell technology may significantly change the future of fertility care. Further intensive study combined with cautious clinical translation will be necessary to guarantee the safety and the effectiveness of these new therapies.
